# Amygdala's T1-weighted image radiomics outperforms volume for differentiation of anxiety disorder and its subtype

**DOI:** 10.3389/fpsyt.2023.1091730

**Published:** 2023-02-24

**Authors:** Qingfeng Li, Wenzheng Wang, Zhishan Hu

**Affiliations:** ^1^Shanghai Mental Health Center, Shanghai Jiao Tong University School of Medicine, Shanghai, China; ^2^State Key Laboratory of Cognitive Neuroscience and Learning, Beijing Normal University, Beijing, China

**Keywords:** anxiety disorder, generalized anxiety disorder, magnetic resonance imaging, amygdala, radiomics

## Abstract

**Introduction:**

Anxiety disorder is the most common psychiatric disorder among adolescents, with generalized anxiety disorder (GAD) being a common subtype of anxiety disorder. Current studies have revealed abnormal amygdala function in patients with anxiety compared with healthy people. However, the diagnosis of anxiety disorder and its subtypes still lack specific features of amygdala from T1-weighted structural magnetic resonance (MR) imaging. The purpose of our study was to investigate the feasibility of using radiomics approach to distinguish anxiety disorder and its subtype from healthy controls on T1-weighted images of the amygdala, and provide a basis for the clinical diagnosis of anxiety disorder.

**Methods:**

T1-weighted MR images of 200 patients with anxiety disorder (including 103 GAD patients) as well as 138 healthy controls were obtained in the Healthy Brain Network (HBN) dataset. We extracted 107 radiomics features for the left and right amygdala, respectively, and then performed feature selection using the 10-fold LASSO regression algorithm. For the selected features, we performed group-wise comparisons, and use different machine learning algorithms, including linear kernel support vector machine (SVM), to achieve the classification between the patients and healthy controls.

**Results:**

For the classification task of anxiety patients vs. healthy controls, 2 and 4 radiomics features were selected from left and right amygdala, respectively, and the area under receiver operating characteristic curve (AUC) of linear kernel SVM in cross-validation experiments was 0.6739±0.0708 for the left amygdala features and 0.6403±0.0519 for the right amygdala features; for classification task for GAD patients vs. healthy controls, 7 and 3 features were selected from left and right amygdala, respectively, and the cross-validation AUCs were 0.6755±0.0615 for the left amygdala features and 0.6966±0.0854 for the right amygdala features. In both classification tasks, the selected amygdala radiomics features had higher discriminatory significance and effect sizes compared with the amygdala volume.

**Discussion:**

Our study suggest that radiomics features of bilateral amygdala potentially could serve as a basis for the clinical diagnosis of anxiety disorder.

## 1. Introduction

As a common brain and behavioral disorder (1), anxiety disorder manifests primarily as excessive fear, worry and avoidance, leading to severe emotional distress, physical illness, cognitive, and behavioral impairments, which further impairs social functioning and quality of life (2). Generalized anxiety disorder (GAD) is a common subtype of anxiety disorder, which is characterized by chronic, excessive anxiety, and worry accompanied by somatic symptoms such as restless, muscle tension, cardiopalmus, and sleep disturbance. The diagnosis of anxiety disorder and its subtypes mainly bases on the presenting symptom, which is subjective and is heavily influenced by the experience of psychiatrist. Biomarkers from neuroimaging, genetics, neurochemistry, and neurophysiology are in critical need for more precise identification of patients with anxiety disorder.

Amygdala plays an important role in the development of anxiety disorder and its subtypes (3, 4). A resting state fMRI study found that adult GAD patients exhibited decreased amygdala sub-regions functional connectivity with the cingulate gyrus insula (5). In addition, numerous structural MR imaging studies have revealed alterations in the volume of the amygdala and its microstructures in patients with anxiety disorder and its subtypes (6–10).

The T1-weighted structural magnetic resonance (MR) imaging is necessary for the spatial registration of other MRI scan. Using the T1-weighted MR imaging only as a biomarker for mental disorders would save a lot of time. Radiomics is a candidate technique to achieve this. Radiomics uses different automated feature extraction algorithms to transform medical images to multi-dimensional advanced quantitative imaging features with high throughput (11, 12). It can be used to explore inherent relationships between image features and clinical diagnosis and symptom presentation. Radiomics was first used in the evaluation of tumor-like diseases (13, 14), and has recently been applied to investigate neurodegenerative diseases (15–18) and psychiatric disorders (19–22).

The T1-weighted MR images are also suitable for radiomics analysis. Previous studies have reported that radiomics features of T1-weighted MR images can be used to distinguish mental disorders such as schizophrenia (19), panic disorder (23), Parkinson's disease with depression (24), and temporal lobe epilepsy (25). Yet, to the best of our knowledge, this technique has not been used for the detection of anxiety disorders.

In this study, we performed radiomic analysis on the high-resolution T1-weighted MR images of bilateral amygdala. Using radiomics features of bilateral amygdala extracted from T1-weighted MR images, we aim to evaluate their feasibility in differentiating anxiety disorders and one of its subtypes (i.e., GAD) from the healthy population.

## 2. Material and methods

### 2.1. Dataset

We analyzed dataset from Child Mind Institute Healthy Brain Network (HBN) (26). The HBN protocol consists of four 3-h sessions collecting general information, behavioral measures, diagnostic assessments, and neuroimaging data. Details of the data acquisition were provided in HBN webpages.^1^ Psychiatric diagnoses were assessed and reported by clinicians according to DSM-5 criteria (27). Among 2,743 subjects in release 1–9 with T1-weighted MR images, we restricted inclusion to participants with diagnosis of anxiety disorder. These patients were further categorized into separation anxiety, specific phobia, GAD, social anxiety and other specified anxiety disorders. Considering the sample size, we selected GAD as a typical subtype of anxiety disorder for our study. After the data quality control (see “Processing” section), 338 participants with 138 healthy controls (HC), 200 patients with anxiety disorders (include 103 GAD patients) were included for further analysis. We randomly sampled 2/3 of the above data into training set and the rest as test set. Feature selection were performed on the training set, and the test set was used as independent validation data to avoid data leakage (Table 1). Note that feature selection, and subsequent machine learning experiments were performed independently for the Anxiety vs. HC and GAD vs. HC tasks.

**Table 1:** Demographics of participants.

	**Training set**			**Test set**			**Training set**			**Test set**		
	**Anxiety disorder**	**HC**	***p*** **-value**	**Anxiety disorder**	**HC**	***p*** **-value**	**GAD**	**HC**	***p*** **-value**	**GAD**	**HC**	***p*** **-value**
Number of subjects	129	96	–	71	42	–	73	96	–	30	42	–
Age (mean ± std)	11.415 ± 2.838	12.087 ± 3.564	0.1311^a^	12.066 ± 3.297	12.008 ± 2.605	0.9228^a^	12.488 ± 3.813	11.415 ± 2.838	0.0386^a^	12.549 ± 2.88	12.008 ± 2.605	0.4145^a^
Gender (male/female)	70/59	46/50	0.5884^b^	34/37	17/25	0.8585^b^	42/31	46/50	0.6384^b^	15/15	17/25	1.0^b^
Site (SI/RU/CUNY/CBIC)	13/53/4/59	28/41/1/26	0.6944^b^	9/26/4/32	18/17/0/7	0.7743^b^	7/26/3/37	28/41/1/26	0.6404^b^	3/12/2/13	18/17/0/7	0.8661^b^

STD, standard deviation; ^a^p-value was calculated using two-sample student's t-test; ^b^p-value was calculated using Chi-square test.

### 2.2. Processing

Quality control using Computational Anatomy Toolbox 12 (CAT12) (28) was performed on T1-weighted MR images. CAT 12 includes various image quality control options, include image resolution, noise, bias field, and weighted overall image quality. Subjects with weighted overall image quality scores of “C+” or lower level were excluded. The remained images were further pre-processed using the standard FreeSurfer recon-all pipeline (version 6.0.0) (29). A probabilistic subcortical structure atlas (i.e., aseg atlas) (30) was used to generate an automated segmentation of bilateral amygdala in native space. The segmentation results were checked by visual inspection.

### 2.3. Radiomics feature extraction

**Figure 1:**
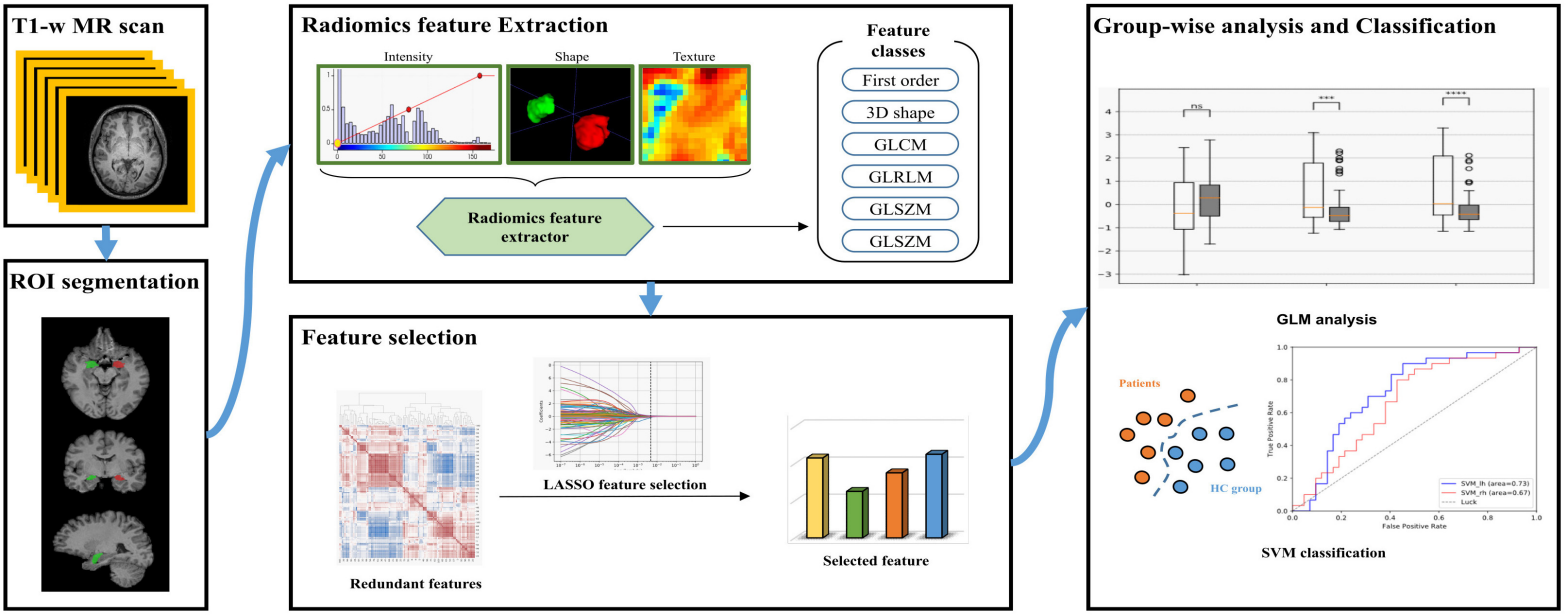
The workflow of this study.

The workflow of our study is shown in Figure 1. PyRadiomics (version 3.0.1) (31), an open-access Python toolkit, was used to extract radiomics features. Radiomics features were calculated using T1-weighted images of left or right amygdala, respectively, which included 18 first-order statistics features, 14 3D shape-based features, 24 gray level co-occurrence matrix (GLCM) features, 16 gray level run length matrix (GLRLM) features, 16 gray level size zone matrix (GLSZM) features, five neighboring gray tone difference matrix (NGTDM) features and 14 gray level dependence matrix (GLDM) features (Supplementary Table 7). In specific, the first-order statistics describes the distribution of voxel intensities within the image region defined by the mask through commonly used and basic metrics. 3D shape-based features are descriptors of the 3D size and shape of the ROI, i.e., amygdala, which are independent from the gray-level intensity distribution in the ROI. GLCM obtains the co-occurrence matrix by counting the probability of the occurrence of pixel pairs in different directions and displacement vectors. It describes the complexity of the lesion, the level variation, and the degree of texture thickness (32, 33). GLRLM obtains the length matrix by calculating the probability of the pixels appearing repeatedly in succession with different directions and displacement vectors, and describes the complexity of the lesion, the level variation and the degree of texture thickness (34). GLSZM quantifies gray level zones in an image (a gray level zone is defined as the number of connected voxels that share the same gray level intensity) (35). NGTDM quantifies the difference between a gray value and the average gray value of its neighbors (36). LDM quantifies gray level dependencies in an image (a gray level dependency is defined as the number of connected voxels that are dependent on the center voxel) (37).

### 2.4. Statistical analysis

#### 2.4.1. Feature selection

**Figure 2:**
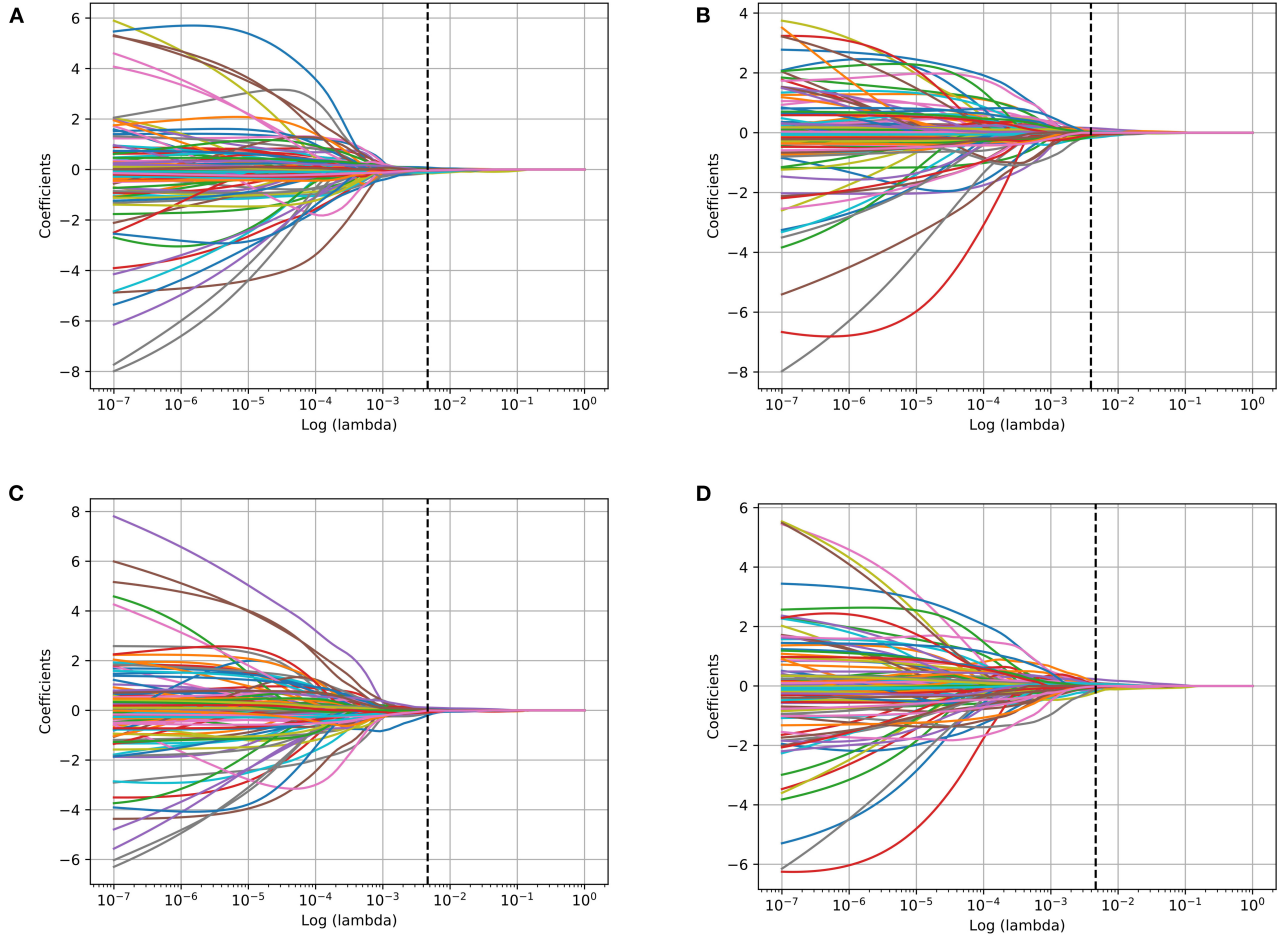
Bilateral amygdala radiomics feature coefficients-lambda graph of the LASSO dimensionality reduction of different tasks. **(A)** Left amygdala features in Anxiety disorder vs. HC task; **(B)** right amygdala features in Anxiety disorder vs. HC task; **(C)** left amygdala features in GAD vs. HC task; **(D)** right amygdala features in GAD vs. HC task.

The range of radiomics features were rescaled *via* z-score normalization. Feature selection was performed on the training dataset from the left and right amygdala, respectively, using the least absolute shrinkage and selection operator (LASSO) regression model with 10-fold cross-validation (Figure 2).

#### 2.4.2. General linear model

Group differences regarding the selected features were tested on the test data. Age, gender and total intracranial volume (TIV) information were also modeled into the GLM. In addition, the volume of the amygdala was also involved in group-wise comparison as a reference to assess the effect of radiomics features. The *p*-values were corrected using Benjamini-Hochberg false discovery rate (FDR) correction method. Further Cohen's d (38) was used to measure the effect size of the difference between patients and HC groups.

#### 2.4.3. Machine learning

Further, linear kernel support vector machine (SVM) (39), a classic machine learning model was used to classify the diagnostic groups. In addition, to verify the performance of radiomics features on different machine learning algorithms, we also used four other effective algorithms, including Radial Basis Function (RBF) kernel SVM, random forest (40), extreme gradient boosting (XGBoost) (41), and Gradient Boosting Decision Tree (GBDT) (42). Details of the parameters of the above algorithms are shown in Table 2. For the radiomics features and volume metric, we also further tried to train models by combining features from the left and right amygdala to verify whether such operation could improve the classification performance. In specific, we used 5-fold cross-validation approach for model validation, and models were trained and tested using the abovementioned selected radiomics features. The model performance was evaluated by the area under curve (AUC) of the receiver operator curve (ROC) for the classification of diagnostic groups.

**Table 2:** Details of the parameters of the used machine learning algorithms.

**Algorithm**	**Parameter name**	**Parameter setting**
Linear kernel SVM	C	1
	Kernel	Linear kernel
	Tolerance	1e-3
RBF kernel SVM	C	1
	Kernel	RBF kernel
	Tolerance	1e-3
Random forest	Estimators number	100
	Criterion	Gini index
	Minimal sample split	2
	Minimal samples leaf	1
XGBoost	Base score	0.5
	Gamma	0
	Learning rate	0.1
	Maximum depth	10
	Estimators number	100
GBDT	Loss	Deviance
	Learning rate	0.1
	Estimators number	100
	Criterion	Friedman mean squared error

SVM, support vector machine; RBF, Radial basis function; XGBoost, extreme gradient boosting; GBDT, Gradient boosting decision tree (GBDT).

## 3. Results

### 3.1. Anxiety disorder vs. HC radiomics feature analysis

Using 10-fold LASSO regression model, we selected 2-dimensional features (i.e., small dependence emphasis and small dependence high gray level emphasis) for the left amygdala, and 4-dimensional features (i.e., maximum 2D diameter column, interquartile range, small dependence emphasis, and gray level non-uniformity normalized) for the right amygdala.

### 3.2. Anxiety disorder vs. HC radiomics feature group-wise comparison

**Figure 3:**
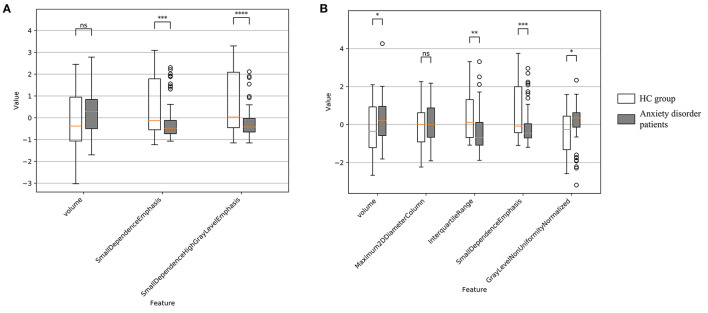
Feature distribution and group-wise comparison results on selected radiomics features of Anxiety disorder vs. HC task. **(A)** Results of left amygdala radiomics features; **(B)** results of right amygdala radiomics features. Points are outliers in the box plot. NS, not significant; *p ≤ 0.05; **p ≤ 0.01; ***p ≤ 0.001; ****p ≤ 0.0001.

For left amygdala, results of group-wise comparison reveals that there were significant differences between anxiety disorder patients and HC group on two selected radiomics features (Figure 3A). As a comparison, no significant difference in left amygdala volume was found between anxiety disorder patients and HC group. The absolute values of the effect sizes of the two radiomics features were also larger than the amygdala volume. For the right amygdala, three radiomics features (i.e., interquartile range, small dependence emphasis, and gray level non-uniformity normalized) and amygdala volume were significantly different between anxiety disorder patients and HC group, and results of the interquartile range and small dependence emphasis was more significant than amygdala volume in group-wise comparison (Figure 3B). In addition, the values of the effect size of the interquartile range, small dependence emphasis, and gray level non-uniformity normalized were also larger than the amygdala volume (Table 3).

**Table 3:** Effect sizes of selected radiomics features and bilateral amygdala volume in anxiety disorder vs. HC task.

**Hemisphere**	**Feature class**	**Feature name**	**Effect size**
Left	Volume	Amygdala volume	0.3392
	GLDM	Small dependence emphasis	0.7587
		Small dependence high gray level emphasis	0.9016
Right	Volume	Amygdala volume	0.42
	3D shape	Maximum 2D diameter column	0.3146
	First order	Interquartile range	0.6028
	GLDM	Small dependence emphasis	0.8085
	GLRLM	Gray level non-uniformity normalized	0.4667

### 3.3. Anxiety disorder vs. HC classification

**Figure 4:**
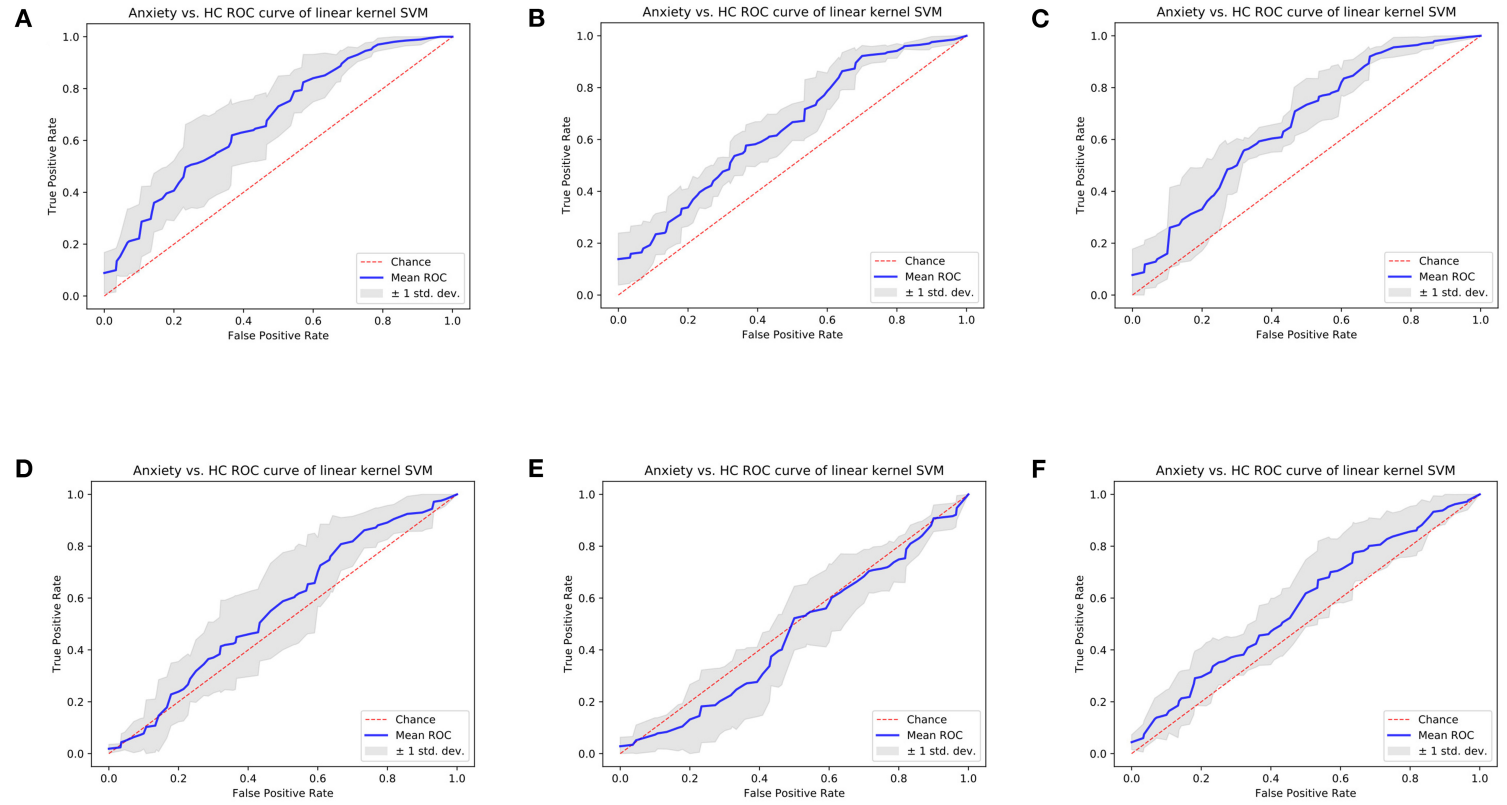
Cross-validation ROC curves of linear kernel SVM model of Anxiety disorder vs. HC classification task. **(A)** Model trained using left amygdala radiomics features, **(B)** model trained using right amygdala radiomics features, **(C)** model trained using bilateral amygdala radiomics features, **(D)** model trained using left amygdala volume, **(E)** model trained using right amygdala volume, and **(F)** model trained using bilateral amygdala volume.

Results of cross-validation experiments showed that the linear kernel SVM models trained separately using selected left/right amygdala radiomics features achieved the classification of anxiety disorder vs. HC. Specifically, SVM trained using two-dimension left amygdala radiomics features achieved classification AUC of 0.6739, and the SVM model trained using four-dimension right amygdala radiomics features achieved classification AUC of 0.6403 (Figures 4A, B, Supplementary Tables 1, 2). For the left/right amygdala, the classification performance of various machine learning algorithms trained with radiomics features were higher than the performance of classifiers trained with amygdala volume (Figures 4A, B, D, E, Supplementary Tables 1–4). Combining features from the left and right amygdala to train machine learning models did not result in a significant improvement in classification performance, but the performance of machine learning models trained by combining radiomics features were still higher than the performance of models trained by combining volume metrics (Figures 4C, F, Supplementary Tables 5, 6).

### 3.4. GAD vs. HC radiomics feature analysis

7-dimensional features (i.e., maximum 2D diameter column, mean absolute deviation, cluster prominence, cluster tendency, small dependence high gray level emphasis, short run high gray level emphasis, and small area high gray level emphasis) for the left amygdala, and three-dimensional features (i.e., maximum 2D diameter column, interquartile range, and cluster tendency) for the right amygdala were selected using 10-fold LASSO regression model.

### 3.5. GAD vs. HC radiomics feature group-wise comparison

**Figure 5:**
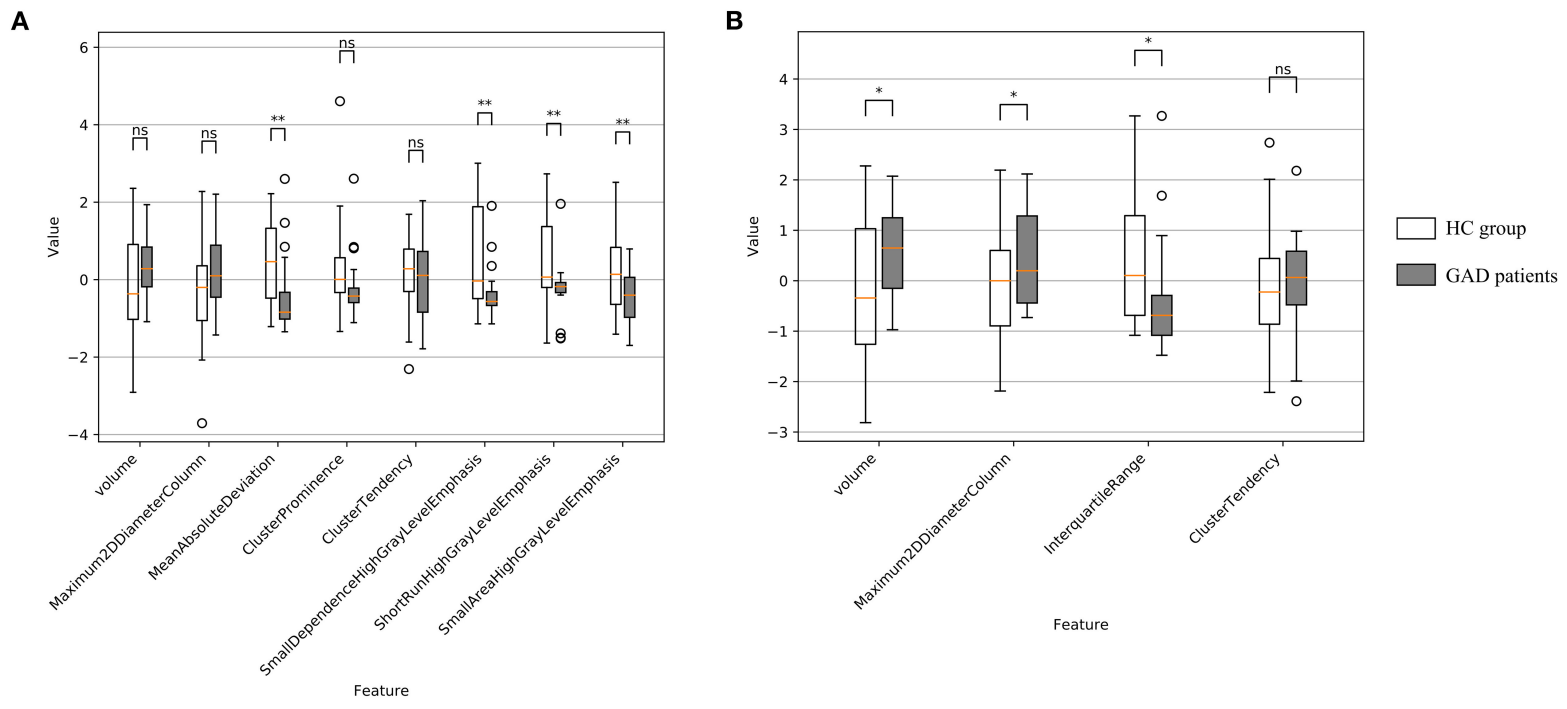
Feature distribution and group-wise comparison results on selected radiomics features of GAD vs. HC task. **(A)** Results of left amygdala radiomics features and **(B)** results of right amygdala radiomics features. Points are outliers in the box plot. ns: not significant; *: *p*-value ≤ 0.05; **: *p*-value ≤ 0.01.

For left amygdala, results of group-wise comparison reveals that there were significant differences between anxiety disorder patients and HC group on 4 selected radiomics features (i.e., mean absolute deviation, small dependence high gray level emphasis, short run high gray level emphasis, and small area high gray level emphasis) (Figure 5A). As a comparison, no significant difference in left amygdala volume was found between anxiety disorder patients and HC group. The absolute values of the effect sizes of five radiomics features (i.e., mean absolute deviation, cluster prominence, small dependence high gray level emphasis, short run high gray level emphasis, and small area high gray level emphasis) were also larger than the amygdala volume. For the right amygdala, two radiomics features (i.e., maximum 2D diameter column, and interquartile range) and amygdala volume were significantly different between anxiety disorder patients and HC group (Figure 5B). In addition, the value of the effect size of the interquartile range was larger than the amygdala volume (Table 4).

**Table 4:** Effect sizes of selected radiomics features and bilateral amygdala volume in GAD vs. HC task.

**Hemisphere**	**Feature class**	**Feature name**	**Effect size**
Left	Volume	Amygdala volume	0.4783
	3D shape	Maximum 2D diameter column	0.4421
	First order	Mean absolute deviation	0.9455
	GLCM	Cluster prominence	0.5997
		Cluster tendency	0.2273
	GLDM	Small dependence high gray level emphasis	0.9212
	GLRLM	Short run high gray level emphasis	0.801
	GLSZM	Small area high gray level emphasis	0.7675
Right	Volume	Amygdala volume	0.6312
	3D shape	Maximum 2D diameter column	0.5661
	First order	Interquartile range	0.757
	GLCM	Cluster tendency	0.1039

### 3.6. GAD vs. HC classification

**Figure 6:**
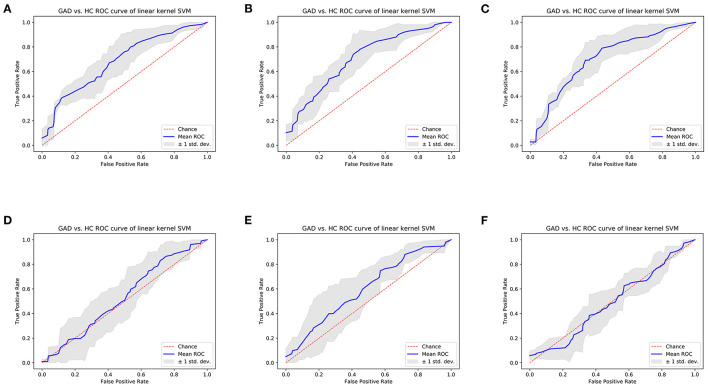
Cross-validation ROC curves of linear kernel SVM model of GAD vs. HC classification task. **(A)** Model trained using left amygdala radiomics features; **(B)** model trained using right amygdala radiomics features; **(C)** model trained using bilateral amygdala radiomics features; **(D)** model trained using left amygdala volume; **(E)** model trained using right amygdala volume; **(F)** model trained using bilateral amygdala volume.

Results showed that the SVM models trained separately using selected left/right amygdala radiomics features achieved the classification of anxiety disorder vs. HC. Specifically, SVM trained using seven-dimension left amygdala radiomics features achieved classification AUC of 0.6755, and the SVM model trained using three-dimension right amygdala radiomics features achieved classification AUC of 0.6966, which were higher than the performance of classifiers trained with amygdala volume (Figures 6A, B, D, E, Supplementary Tables 1–4). Combining features from the left and right amygdala to train machine learning models did not result in a significant improvement in classification performance, but the performance of machine learning models trained by combining radiomics features were still higher than the performance of models trained by combining volume metrics (Figures 6C, F, Supplementary Tables 5, 6).

## 4. Discussion

Our study indicated that patients with anxiety disorders and GAD showed abnormalities in the left/right amygdala radiomics features compared with the HC group. Group-wise comparison revealed that abnormalities of some radiomics features were more significant than amygdala volume. Our study is a prospective research to evaluate the feasibility of differentiating anxiety disorders and one of its subtypes (i.e., GAD) from the healthy population using radiomics features of bilateral amygdala extracted from T1-weighted MR images.

Radiomics analysis has been applied to some neural psychiatric disorders. A study has found that radiomics features extracted from the hippocampus structure reflect high-order imaging patterns and heterogeneity characteristics of microstructure in hippocampus in AD patients (43). A radiomics study of autism spectrum disorder has found significant differences in the texture features in the right hippocampus, corpus callosum, cerebellar white matter, and left choroid plexus between patients and controls (44). However, radiomics studies of anxiety disorder and its subtypes using T1-weighted structural MR images are still lacking. Structural MR imaging studies have revealed alterations in the volume of the amygdala in patients with anxiety disorder and its subtypes (6–9). In our study, we used radiomics technique to further analyze the abnormalities of the amygdala in anxiety disorders and its subtype (i.e., GAD). Radiomics features selected by LASSO regression model reflect the gray value distribution, spatial heterogeneity, texture characteristics and other microstructural information.

For anxiety disorders, there were 2 selected radiomics features of left amygdala, i.e., small dependence emphasis and small dependence high gray level emphasis are GLDM parameters. According to existing study (37), gray level dependency is defined as the number of connected voxels that are dependent on the center voxel, and small dependence emphasis is a measure of the distribution of small dependencies, with a lower value indicative of greater dependence and more homogeneous textures of left amygdala of anxiety disorder patients compared with HC group. Small dependence high gray level emphasis measures the joint distribution of small dependence with higher gray-level values, with a lower value indicating a smaller concentration of high gray-level values in the image. For right amygdala, there were 4 selected radiomics features, including maximum 2D diameter column, interquartile range, small dependence emphasis, and gray level non-uniformity normalized. Maximum 2D diameter (Column) is defined as the largest pairwise Euclidean distance between ROI surface mesh vertices in the row-slice (usually the coronal) plane. Interquartile range is defined as difference between 25th and 75th percentile of the gray level intensity within the ROI. Gray level non-uniformity normalized measures the variability of gray-level intensity values in the image, with a greater value indicating a smaller similarity in intensity values. The above features indicate structural and textural heterogeneity in the right amygdala in patients with anxiety disorder.

For GAD, seven radiomics features of left amygdala were selected, including maximum 2D diameter column, mean absolute deviation, cluster prominence, cluster tendency, small dependence high gray level emphasis, short run high gray level emphasis, and small area high gray level emphasis. Mean absolute deviation is the mean distance of all intensity values from the mean value of the gray level intensity values within the ROI. Cluster prominence and cluster tendency are GLCM parameters (32). A lower values of cluster prominence implies less asymmetry of the GLCM, and cluster tendency is a measure of groupings of voxels with similar gray-level values. Short run high gray level emphasis measures the joint distribution of shorter run lengths with higher gray-level values (34), with a lower value indicating a smaller concentration of high gray-level values in the image. Small area high gray level emphasis measures the proportion in the image of the joint distribution of smaller size zones with higher gray-level values, with a lower value indicating a smaller proportion of higher gray-level values of small size zone in the image (35). For right amygdala, there were 3 selected features, i.e., maximum 2D diameter column, interquartile range, and cluster tendency. These features extracted from the left/right amygdala structure reflect high-order imaging patterns and heterogeneity characteristics of microstructure in amygdala in GAD patients.

It is worth noting that significant results in group-wise comparison were not observed on some LASSO-selected radiomics features (e.g., maximum 2D diameter column of right amygdala and cluster tendency of bilateral amygdala). As a machine learning method, LASSO integrates each feature dimension to assess feature importance, while the statistical method of group-wise comparison performs hypothesis testing independently for a specific feature dimension. Therefore, the possible reason for the above experimental results is that certain features that do not differ significantly between patients and healthy people are important for the machine learning task. The above reason can also explain the experimental results related to volume metrics. Although right amygdala volume was significantly different in both Anxiety vs. HC and GAD vs. HC group comparisons (Figures 3B, 5B), satisfactory classification results could not be obtained from machine learning classifiers that trained using right amygdala volume (Figures 4E, 6F, Supplementary Tables 4, 6). This may due to the fact that such differences may not necessarily valid for training machine learning models, e.g., SVM.

Existing studies have used radiomics features for machine learning-based neuropsychiatric disorders classification. A study (20) identified 30 radiomics features of corpus callosum to differentiate participants with schizophrenia from HCs using Bayesian optimized model. Another study (45) used texture features based on GLCM to separate autism spectrum disorder and development control subjects using SVM and random forest classifiers. In a recent study (46), logistic regression analysis was performed to build classification models based on amygdala radiomics features for Alzheimer's disease and amnestic mild cognitive impairment, and achieved an AUC of 0.93 for AD vs. NC classification, an AUC of 0.84 for AD vs. aMCI classification, and an AUC of 0.80 for aMCI vs. NC classification. However, there are still lack of studies on radiomics-based anxiety disorder-related classification. In our study, SVM classification experiments have demonstrated that selected radiomics features of the left/right amygdala can be used to separate patients with anxiety disorder and GAD from HC group, and using radiomics features of amygdala for classification is better than using amygdala volume. In addition, for both anxiety disorder vs. HC and GAD vs. HC classification tasks, SVM classifiers trained using radiomics features of left amygdala achieved higher AUC than that of right amygdala, which implies that the microstructural changes associated with anxiety are greater in the left amygdala compared with the right amygdala.

Exist studies have revealed alterations in the volume of the amygdala in patients with anxiety disorder and its subtypes. Research on amygdala subregional structure suggests that microstructural information of amygdala is also associated with anxiety-related disorders (10). Radiomics features could reflect high-order imaging patterns and heterogeneity characteristics of microstructure in bilateral amygdala. According to group-wise comparison experiments, the differences between patients and HC group in most selected radiomics features were more significant than the amygdala volume. In addition, the absolute values of the effect sizes of most selected radiomics features were larger than the amygdala volume. Our study suggests bilateral amygdala radiomics features could serve as more effective neuroimaging biomarkers, compared with amygdala volume, for identifying patients with anxiety disorders and GAD.

There were several limitations in our study. Firstly, there are many subtypes of anxiety disorder, including social anxiety, separation anxiety, etc. Limited by sample size, only GAD was selected as an example for anxiety disorder subtype in our study. Secondly, a complete 1:1 match in age, sex and site ratio had not been achieved. In future works, we will collect data of other anxiety disorder subtypes scanned from multiple scanners, and further evaluate relationship between amygdala radiomics features and behavioral information.

In summary, our study observed that compared with amygdala volume, bilateral amygdala radiomics features could serve as more effective neuroimaging biomarkers for identifying patients with anxiety disorders and GAD. Moreover, we used machine learning method to evaluate the feasibility of differentiating patients of anxiety disorders and GAD from the healthy people using radiomics features of bilateral amygdala extracted from T1-weighted MR images, thus providing effective biomarkers for the clinical diagnosis of anxiety disorders.

## Data availability statement

Publicly available datasets were analyzed in this study. This data can be found at: Child Mind Institute Healthy Brain Network (HBN) dataset: http://fcon_1000.projects.nitrc.org/indi/cmi_healthy_brain_network.

## Ethics statement

The studies involving human participants were reviewed and approved by Chesapeake Institutional Review Board. Written informed consent to participate in this study was provided by the participants' legal guardian/next of kin.

## Author contributions

QL, WW, and ZH designed the experiments and drafted and reviewed the manuscript. QL and WW performed the experiments and analyzed the data and interpreted the results. All the authors read and approved the final version of the manuscript.

## Footnotes

^1^ http://fcon_1000.projects.nitrc.org/indi/cmi_healthy_brain_network
