# Supplementary material for: Amygdala's T1-weighted image radiomics outperforms volume for differentiation of anxiety disorder and its subtype
Source: Front Psychiatry. 2023 Feb 24;14:1091730. doi: 10.3389/fpsyt.2023.1091730 (PMC10001895; doi:10.3389/fpsyt.2023.1091730)
Supplement: Supplementary file 1 [file Table_1.docx]

Supplementary Material

# Supplementary Figures and Tables

## Supplementary Tables

**Supplementary Table 1.** Cross-validation classification performance of different machine learning models on Anxiety disorder vs. HC task and GAD vs. HC task using left amygdala radiomics features. ACC: accuracy; SEN: sensitivity; SPE: specificity; AUC: area under ROC curve; std: standard deviation; SVM: support vector machine; RBF: Radial Basis Function; XGBoost: extreme gradient boosting; GBDT: Gradient Boosting Decision Tree (GBDT).

| Task | Algorithm | ACC (mean±std) | SEN (mean±std) | SPE (mean±std) | AUC (mean±std) |
| --- | --- | --- | --- | --- | --- |
| Anxiety disorder vs. HC | linear kernel SVM | 0.6715±0.0336 | 0.9121±0.0386 | 0.3265±0.0521 | 0.6739±0.0708 |
|  | RBF kernel SVM | 0.6567±0.0255 | 0.8927±0.0424 | 0.3203±0.0628 | 0.6372±0.0425 |
|  | Random forest | 0.5739±0.0179 | 0.6857±0.0330 | 0.4099±0.0519 | 0.5706±0.0498 |
|  | XGBoost | 0.5650±00.0398 | 0.6547±0.0542 | 0.4361±0.0373 | 0.5567±0.0487 |
|  | GBDT | 0.6152±0.0215 | 0.7501±0.0296 | 0.4126±0.0864 | 0.5957±0.0377 |
| GAD vs. HC | linear kernel SVM | 0.6140±0.0541 | 0.5298±0.0716 | 0.6858±0.1092 | 0.6755±0.0615 |
|  | RBF kernel SVM | 0.6055±0.0559 | 0.5480±0.0896 | 0.6575±0.1099 | 0.6801±0.0797 |
|  | Random forest | 0.5890±0.0398 | 0.4697±0.0566 | 0.6857±0.0943 | 0.6648±0.0615 |
|  | XGBoost | 0.6013±0.0585 | 0.4608±0.0677 | 0.7046±0.0634 | 0.6056±0.0496 |
|  | GBDT | 0.6054±0.0796 | 0.5103±0.0549 | 0.6795±0.1214 | 0.6703±0.0608 |

**Supplementary Table 2.** Cross-validation classification performance of different machine learning models on Anxiety disorder vs. HC task and GAD vs. HC task using right amygdala radiomics features. ACC: accuracy; SEN: sensitivity; SPE: specificity; AUC: area under ROC curve; std: standard deviation; SVM: support vector machine; RBF: Radial Basis Function; XGBoost: extreme gradient boosting; GBDT: Gradient Boosting Decision Tree (GBDT).

| Task | Algorithm | ACC (mean±std) | SEN (mean±std) | SPE (mean±std) | AUC (mean±std) |
| --- | --- | --- | --- | --- | --- |
| Anxiety disorder vs. HC | linear kernel SVM | 0.6567±0.0133 | 0.8829±0.0479 | 0.3332±0.0222 | 0.6403±0.0519 |
|  | RBF kernel SVM | 0.6567±0.0163 | 0.8873±0.0404 | 0.3265±0.0259 | 0.6122±0.0504 |
|  | Random forest | 0.5976±0.0723 | 0.7304±0.1200 | 0.4108±0.0622 | 0.5432±0.0649 |
|  | XGBoost | 0.5384±0.0513 | 0.6232±0.0806 | 0.4136±0.0951 | 0.5298±0.0732 |
|  | GBDT | 0.5710±0.0399 | 0.7176±0.0782 | 0.3565±0.0899 | 0.5524±0.0634 |
| GAD vs. HC | linear kernel SVM | 0.6221±0.0581 | 0.6364±0.1042 | 0.6211±0.1150 | 0.6966±0.0854 |
|  | RBF kernel SVM | 0.6059±0.0667 | 0.5481±0.1676 | 0.6637±0.0988 | 0.6415±0.0823 |
|  | Random forest | 0.6182±0.0507 | 0.4984±0.0794 | 0.7106±0.0560 | 06373±0.0479 |
|  | XGBoost | 0.5599±0.0402 | 0.4579±0.1011 | 0.6397±0.0506 | 0.5537±0.0630 |
|  | GBDT | 0.5599±0.0486 | 0.4279±0.0546 | 0.6636±0.0815 | 0.5880±0.0924 |

**Supplementary Table 3.** Cross-validation classification performance of different machine learning models on Anxiety disorder vs. HC task and GAD vs. HC task using left amygdala volume. ACC: accuracy; SEN: sensitivity; SPE: specificity; AUC: area under ROC curve; std: standard deviation; SVM: support vector machine; RBF: Radial Basis Function; XGBoost: extreme gradient boosting; GBDT: Gradient Boosting Decision Tree (GBDT).

| Task | Algorithm | ACC (mean±std) | SEN (mean±std) | SPE (mean±std) | AUC (mean±std) |
| --- | --- | --- | --- | --- | --- |
| Anxiety disorder vs. HC | linear kernel SVM | 0.5918±0.0426 | 1.0000±0.0000 | 0.0000±0.0000 | 0.5526±0.0952 |
|  | RBF kernel SVM | 0.6036±0.0147 | 0.9178±0.0530 | 0.1501±0.0522 | 0.5322±0.0536 |
|  | Random forest | 0.5504±0.0468 | 0.6097±0.0324 | 0.4561±0.1311 | 0.5545±0.0788 |
|  | XGBoost | 0.5621±0.0779 | 0.6619±0.0557 | 0.4112±0.1387 | 0.5447±0.0982 |
|  | GBDT | 0.5769±0.0798 | 0.7223±0.0761 | 0.3612±0.1153 | 0.5623±0.0798 |
| GAD vs. HC | linear kernel SVM | 0.5729±0.0373 | 0.0000±0.0000 | 1.0000±0.0000 | 0.5263±0.1150 |
|  | RBF kernel SVM | 0.5312±0.0264 | 0.2389±0.2120 | 0.7695±0.1938 | 0.5203±0.0718 |
|  | Random forest | 0.5435±0.0512 | 0.4479±0.1132 | 0.6197±0.1006 | 0.5590±0.0381 |
|  | XGBoost | 0.5478±0.0394 | 0.4567±0.1045 | 0.6175±0.0841 | 0.5394±0.0312 |
|  | GBDT | 0.5477±0.0243 | 0.4395±0.0507 | 0.6337±0.0708 | 0.5630±0.0324 |

**Supplementary Table 4.** Cross-validation classification performance of different machine learning models on Anxiety disorder vs. HC task and GAD vs. HC task using right amygdala volume. ACC: accuracy; SEN: sensitivity; SPE: specificity; AUC: area under ROC curve; std: standard deviation; SVM: support vector machine; RBF: Radial Basis Function; XGBoost: extreme gradient boosting; GBDT: Gradient Boosting Decision Tree (GBDT).

| Task | Algorithm | ACC (mean±std) | SEN (mean±std) | SPE (mean±std) | AUC (mean±std) |
| --- | --- | --- | --- | --- | --- |
| Anxiety disorder vs. HC | linear kernel SVM | 0.5918±0.0426 | 1.0000±0.0000 | 0.0000±0.0000 | 0.4523±0.0857 |
|  | RBF kernel SVM | 0.5946±0.0341 | 0.8611±0.0946 | 0.2102±0.1346 | 0.5106±0.0953 |
|  | Random forest | 0.5237±0.0517 | 0.6242±0.0790 | 0.3871±0.0930 | 0.5484±0.0585 |
|  | XGBoost | 0.5237±0.0300 | 0.6334±0.0987 | 0.3733±0.1100 | 0.5247±0.0429 |
|  | GBDT | 0.5205±0.0495 | 0.6740±0.0776 | 0.3031±0.1097 | 0.5153±0.0422 |
| GAD vs. HC | linear kernel SVM | 0.5437±0.0495 | 0.1300±0.1600 | 0.8571±0.1764 | 0.5923±0.1101 |
|  | RBF kernel SVM | 0.5065±0.0471 | 0.2361±0.1323 | 0.7105±0.1481 | 0.4749±0.0278 |
|  | Random forest | 0.5101±0.0412 | 0.4585±0.0690 | 0.5542±0.0766 | 0.5023±0.0485 |
|  | XGBoost | 0.5188±0.0429 | 0.4746±0.0917 | 0.5605±0.0752 | 0.4782±0.0411 |
|  | GBDT | 0.5019±0.0263 | 0.3984±0.0319 | 0.5823±0.0556 | 0.4745±0.0518 |

**Supplementary Table 5.** Cross-validation classification performance of different machine learning models on Anxiety disorder vs. HC task and GAD vs. HC task using bilateral radiomics features. ACC: accuracy; SEN: sensitivity; SPE: specificity; AUC: area under ROC curve; std: standard deviation; SVM: support vector machine; RBF: Radial Basis Function; XGBoost: extreme gradient boosting; GBDT: Gradient Boosting Decision Tree (GBDT).

| Task | Algorithm | ACC (mean±std) | SEN (mean±std) | SPE (mean±std) | AUC (mean±std) |
| --- | --- | --- | --- | --- | --- |
| Anxiety disorder vs. HC | linear kernel SVM | 0.6686±0.0188 | 0.8977±0.0415 | 0.3403±0.0454 | 0.6541±0.0573 |
|  | RBF kernel SVM | 0.6567±0.0210 | 0.8927±0.0424 | 0.3203±0.0591 | 0.6249±0.0520 |
|  | Random forest | 0.6450±0.0311 | 0.7878±0.0493 | 0.4413±0.1007 | 0.6147±0.0331 |
|  | XGBoost | 0.5826±0.0373 | 0.6867±0.0738 | 0.4337±0.0581 | 0.5758±0.0356 |
|  | GBDT | 0.5888±0.0464 | 0.6998±0.0828 | 0.4328±0.0608 | 0.5982±0.0401 |
| GAD vs. HC | linear kernel SVM | 0.6430±0.0413 | 0.6461±0.0586 | 0.6501±0.1000 | 0.6976±0.0608 |
|  | RBF kernel SVM | 0.6264±0.0306 | 0.5686±0.0772 | 0.6792±0.1059 | 0.6614±0.0948 |
|  | Random forest | 0.6179±0.0644 | 0.4852±0.0822 | 0.7230±0.0993 | 0.6620±0.0685 |
|  | XGBoost | 0.5639±0.0681 | 0.4333±0.1002 | 0.6646±0.0872 | 0.5796±0.0585 |
|  | GBDT | 0.5681±0.0489 | 0.4494±0.0772 | 0.6647±0.0940 | 0.6287±0.0478 |

**Supplementary Table 6.** Cross-validation classification performance of different machine learning models on Anxiety disorder vs. HC task and GAD vs. HC task using bilateral amygdala volume. ACC: accuracy; SEN: sensitivity; SPE: specificity; AUC: area under ROC curve; std: standard deviation; SVM: support vector machine; RBF: Radial Basis Function; XGBoost: extreme gradient boosting; GBDT: Gradient Boosting Decision Tree (GBDT).

| Task | Algorithm | ACC (mean±std) | SEN (mean±std) | SPE (mean±std) | AUC (mean±std) |
| --- | --- | --- | --- | --- | --- |
| Anxiety disorder vs. HC | linear kernel SVM | 0.5918±0.0426 | 1.0000±0.0000 | 0.0000±1.0000 | 0.5670±0.0815 |
|  | RBF kernel SVM | 0.6153±0.0256 | 0.9019±0.0300 | 0.2016±0.0661 | 0.5542±0.0821 |
|  | Random forest | 0.5356±0.0227 | 0.6744±0.0837 | 0.3437±0.0783 | 0.5437±0.0639 |
|  | XGBoost | 0.5356±0.0500 | 0.6389±0.0905 | 0.3962±0.0875 | 0.5175±0.0555 |
|  | GBDT | 0.5294±0.0408 | 0.6732±0.0730 | 0.3260±0.0636 | 0.5271±0.0557 |
| GAD vs. HC | linear kernel SVM | 0.5479±0.0445 | 0.1200±0.1470 | 0.8714±0.1591 | 0.4860±0.0754 |
|  | RBF kernel SVM | 0.4979±0.0449 | 0.2190±0.1926 | 0.7274±0.2002 | 0.5017±0.0263 |
|  | Random forest | 0.5186±0.0212 | 0.3906±0.0363 | 0.6184±0.0570 | 0.5309±0.0584 |
|  | XGBoost | 0.5018±0.0403 | 0.3371±0.0836 | 0.6250±0.0523 | 0.4864±0.0452 |
|  | GBDT | 0.5019±0.0721 | 0.3801±0.0721 | 0.5997±0.1121 | 0.5221±0.0358 |

**Supplementary Table 7.** PyRadiomics-extracted 107 radiomics features.

| first-order statistics features | 3D shape-based features | gray level co-occurrence matrix features | gray level run length matrix features | gray level size zone matrix features | neighboring gray tone difference matrix features | gray level dependence matrix features |
| --- | --- | --- | --- | --- | --- | --- |
| firstorder_10Percentile  firstorder_90Percentile  firstorder_Energy  firstorder_Entropy  firstorder_InterquartileRange  firstorder_Kurtosis  firstorder_Maximum  firstorder_MeanAbsoluteDeviation  firstorder_Mean  firstorder_Median  firstorder_Minimum  firstorder_Range  firstorder_RobustMeanAbsoluteDeviation  firstorder_RootMeanSquared  firstorder_Skewness  firstorder_TotalEnergy  firstorder_Uniformity  firstorder_Variance | shape_Elongation  shape_Flatness  shape_LeastAxisLength  shape_MajorAxisLength  shape_Maximum2DDiameterColumn  shape_Maximum2DDiameterRow  shape_Maximum2DDiameterSlice  shape_Maximum3DDiameter  shape_MeshVolume  shape_MinorAxisLength  shape_Sphericity  shape_SurfaceArea  shape_SurfaceVolumeRatio  shape_VoxelVolume | glcm_Autocorrelation  glcm_ClusterProminence  glcm_ClusterShade  glcm_ClusterTendency  glcm_Contrast  glcm_Correlation  glcm_DifferenceAverage  glcm_DifferenceEntropy  glcm_DifferenceVariance  glcm_Id  glcm_Idm  glcm_Idmn  glcm_Idn  glcm_Imc1  glcm_Imc2  glcm_InverseVariance  glcm_JointAverage  glcm_JointEnergy  glcm_JointEntropy  glcm_MCC  glcm_MaximumProbability  glcm_SumAverage  glcm_SumEntropy  glcm_SumSquares | glrlm_GrayLevelNonUniformity  glrlm_GrayLevelNonUniformityNormalized  glrlm_GrayLevelVariance  glrlm_HighGrayLevelRunEmphasis  glrlm_LongRunEmphasis  glrlm_LongRunHighGrayLevelEmphasis  glrlm_LongRunLowGrayLevelEmphasis  glrlm_LowGrayLevelRunEmphasis  glrlm_RunEntropy  glrlm_RunLengthNonUniformity  glrlm_RunLengthNonUniformityNormalized  glrlm_RunPercentage  glrlm_RunVariance  glrlm_ShortRunEmphasis  glrlm_ShortRunHighGrayLevelEmphasis  glrlm_ShortRunLowGrayLevelEmphasis | glszm_GrayLevelNonUniformity  glszm_GrayLevelNonUniformityNormalized  glszm_GrayLevelVariance  glszm_HighGrayLevelZoneEmphasis  glszm_LargeAreaEmphasis  glszm_LargeAreaHighGrayLevelEmphasis  glszm_LargeAreaLowGrayLevelEmphasis  glszm_LowGrayLevelZoneEmphasis  glszm_SizeZoneNonUniformity  glszm_SizeZoneNonUniformityNormalized  glszm_SmallAreaEmphasis  glszm_SmallAreaHighGrayLevelEmphasis  glszm_SmallAreaLowGrayLevelEmphasis  glszm_ZoneEntropy  glszm_ZonePercentage  glszm_ZoneVariance | ngtdm_Busyness  ngtdm_Coarseness  ngtdm_Complexity  ngtdm_Contrast  ngtdm_Strength | gldm_DependenceEntropy  gldm_DependenceNonUniformity  gldm_DependenceNonUniformityNormalized  gldm_DependenceVariance  gldm_GrayLevelNonUniformity  gldm_GrayLevelVariance  gldm_HighGrayLevelEmphasis  gldm_LargeDependenceEmphasis  gldm_LargeDependenceHighGrayLevelEmphasis  gldm_LargeDependenceLowGrayLevelEmphasis  gldm_LowGrayLevelEmphasis  gldm_SmallDependenceEmphasis  gldm_SmallDependenceHighGrayLevelEmphasis  gldm_SmallDependenceLowGrayLevelEmphasis |
